# Supplementary material for: Structural and Interactional Analysis of the Flavonoid Pathway Proteins: Chalcone Synthase, Chalcone Isomerase and Chalcone Isomerase-like Protein
Source: Int J Mol Sci. 2024 May 22;25(11):5651. doi: 10.3390/ijms25115651 (PMC11172311; doi:10.3390/ijms25115651)
Supplement: Supplementary file 1 [file ijms-25-05651-s001.zip › ijms-2974375-supplementary.pdf]

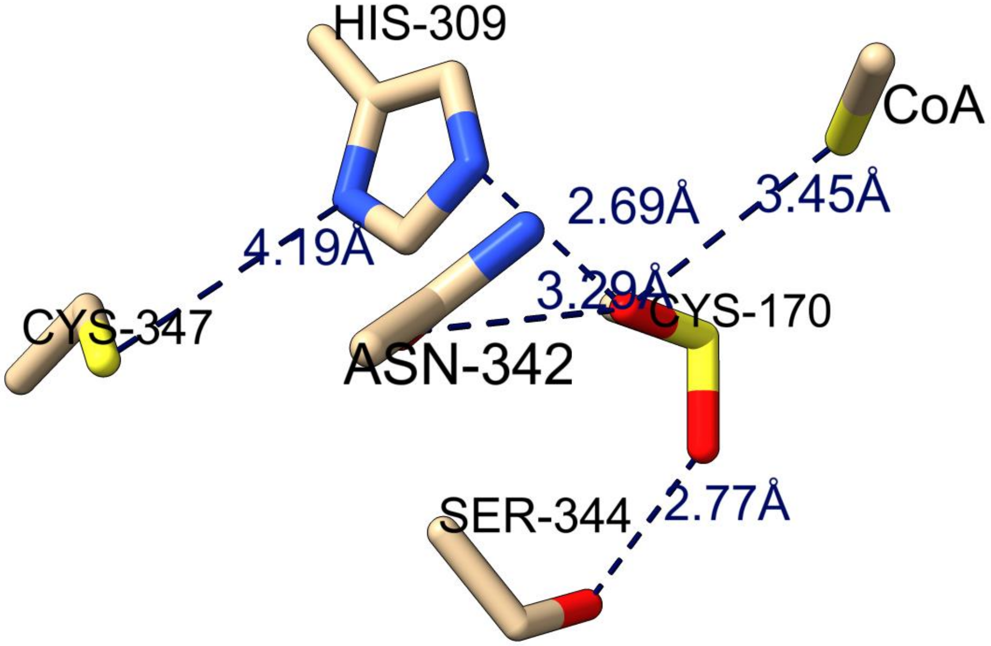

Figure S1. The active site of PvCHS. Two catalytic residues, Cys-167 and His-306 are displayed with two neighboring residues, Ser-341 and Cys-344. The *pKa* value of Cys-167 could be lowered by these interactions. The figure was generated using UCSF ChimeraX.

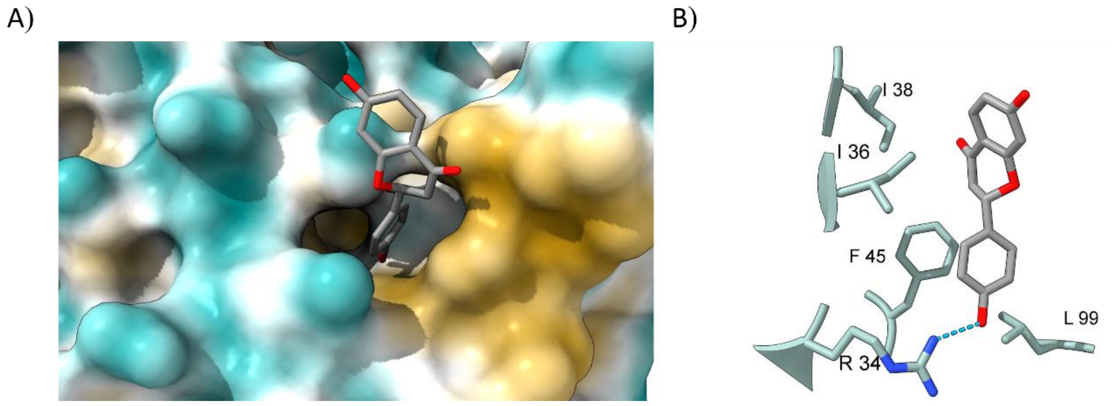

Fig S2. Active site of SbCHI with liquiritigenin. A) Surface diagram of SbCHI with hydrophobic residues shown in yellow and hydrophilic residues in blue. **B)** Active site residues of SbCHI with hydrogen bonds shown using a dotted blue line.

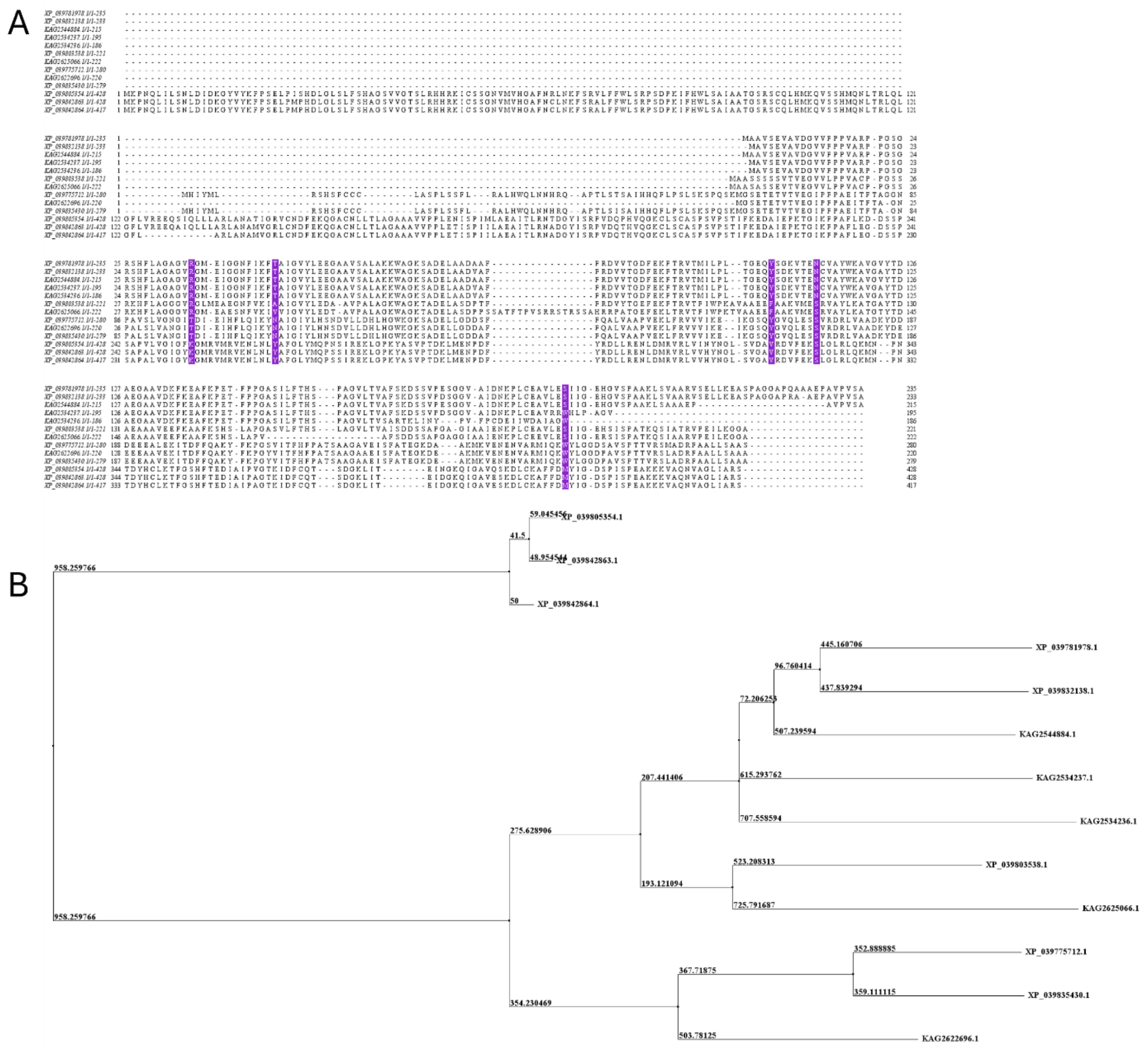

Figure S3. A) A blast search towards PvCHI for the homologs in *Panicum virgatum* was performed using clustal omega. The active site residues of CHI are aligned and shown in purple. B) A distance tree was calculated using Blossum62 method. Graphics and alignment were conducted using Jalivew (University of Dundee, United Kingdom).

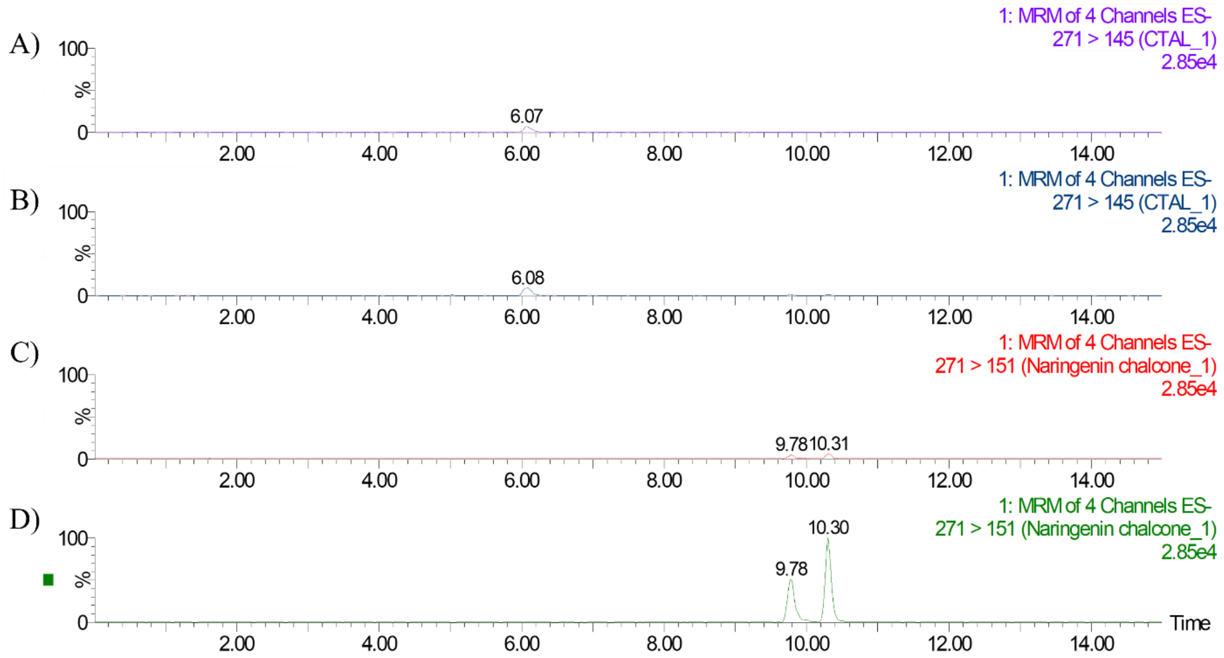

Figure S4. Chromatogram of CHS reaction mixtures with MRM transition analysis. A and B) Transition for *p*-coumaroyltriacetic acid lactone (CTAL) from 271>145 m/z and C and D) Naringenin chalcone and naringenin 275>151 m/z

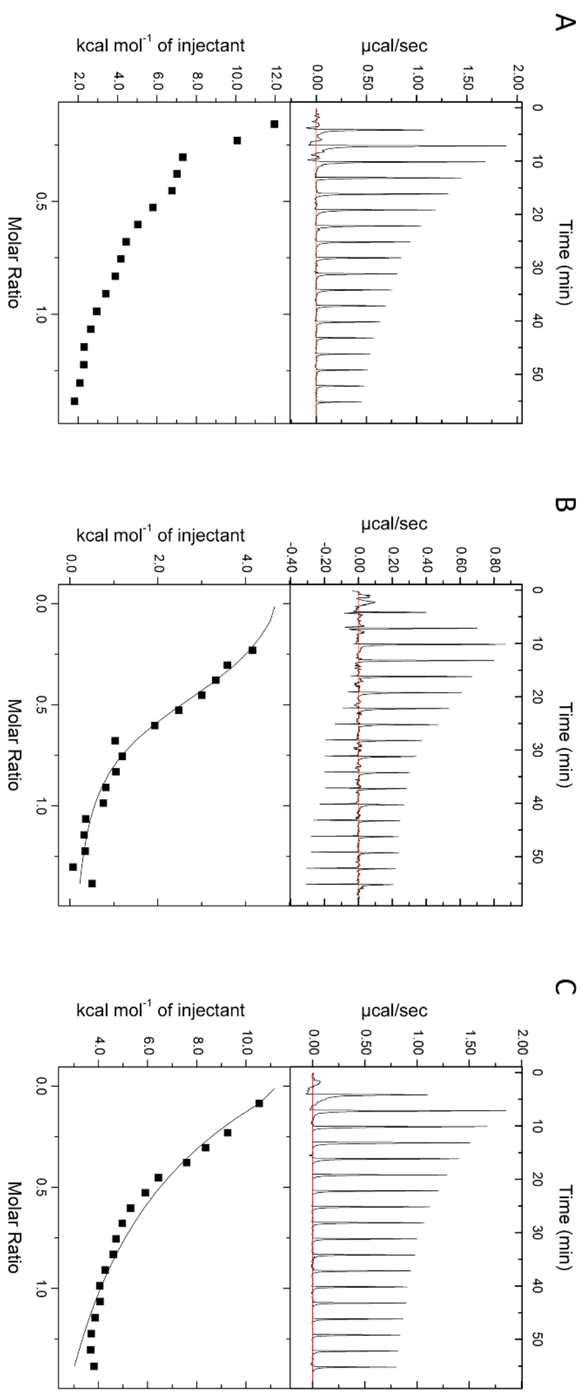

Figure S5. Isothermal Titration Calorimetry of PvCHIL titrated into PvCHS. **A)** was conducted with no substrate, **B)** was conducted with naringenin, **C)** was conducted with naringenin chalcone. Data was evaluated and plots were constructed utilizing Origin 7 (Northampton, MA, USA).

Equation 1: $\Delta Em_{521} = \frac{n}{2}([CHI] + [CHS] + K_d - \sqrt{([CHI] + [CHS] + K_d{}^2) - 4}[CHI][CHS])$

Equation 2: $\Delta Em_{575} = \frac{n}{2}([CHIL] + [CHS] + K_d - \sqrt{([CHIL] + [CHS] + K_d{}^2) - 4}[CHIL][CHS])$

Figure S6. Equations used for calculating of **the** K_d from the FRET experiment.

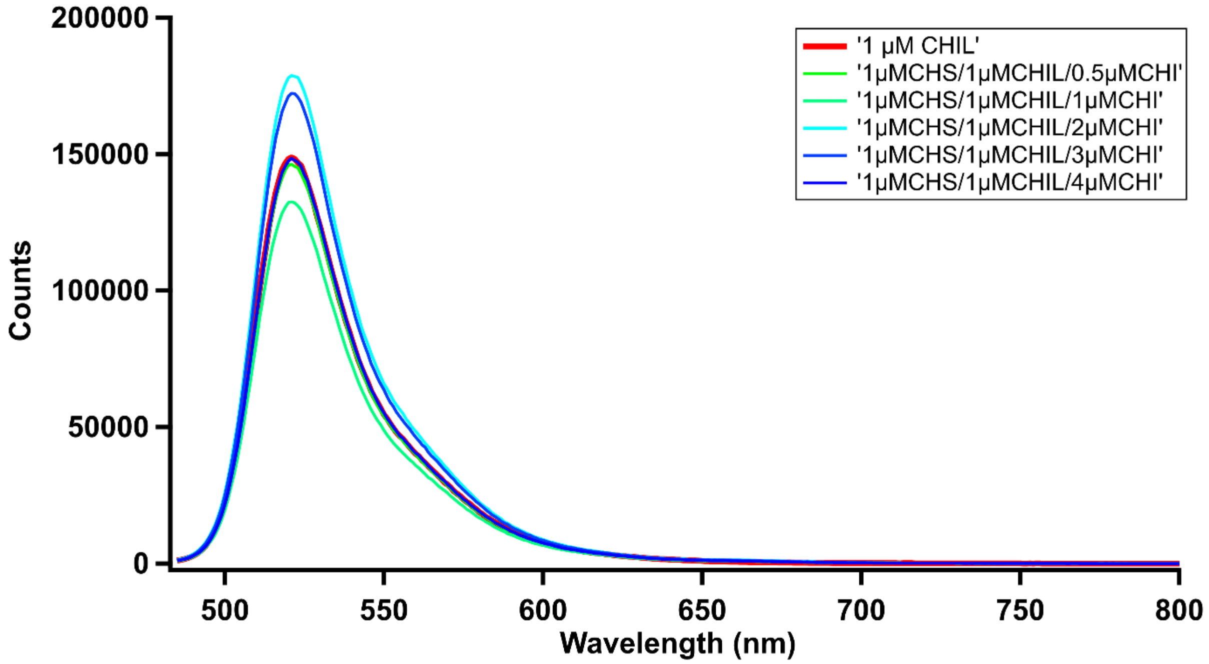

Figure S7. 1 µM of CHIL(ATTO 488) and CHS (ATTO 647N) is held constant throughout each addition of unlabeled CHI. The doner dye was excited at 470nm.

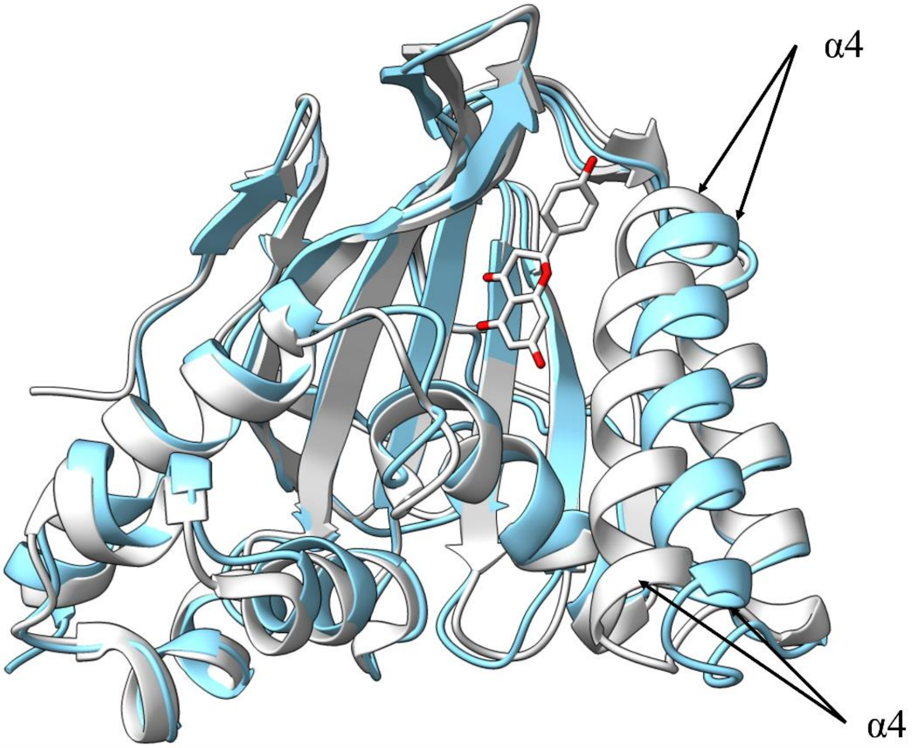

Figure S8. Structural comparison of PvCHI (blue, PDBID:8V8L) and MsCHI (white, PDBID:1EYQ). Naringenin from 1EYQ structure is included and α4 is labelled as it is largely shifted away from the product observed in MsCHI.

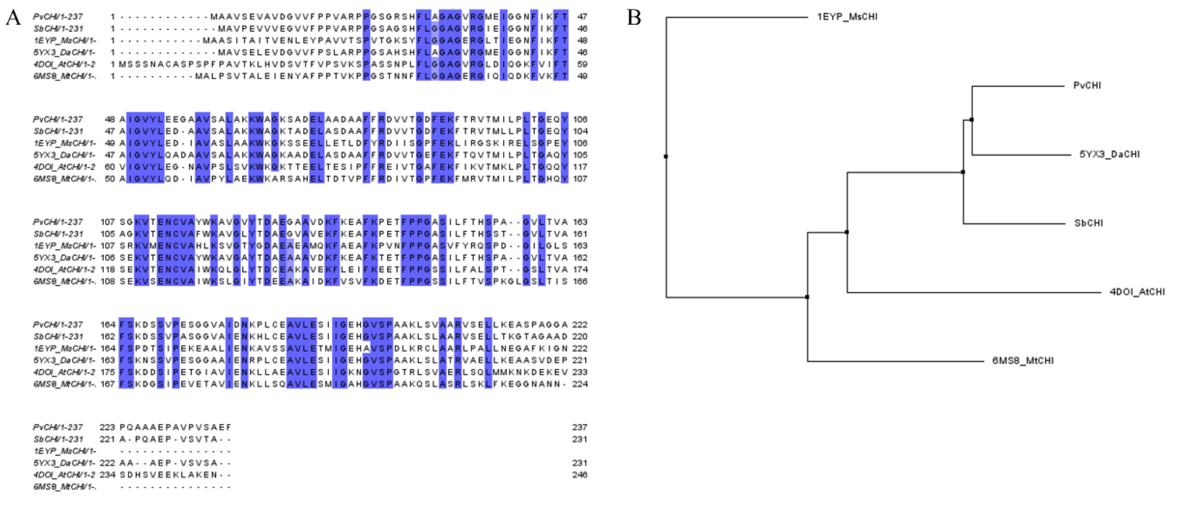

Figure S9. A) The six type-I CHI structures deposited in the Protein Data Bank are aligned with residues with 100% conservation highlighted in purple. B) A distance tree between the six type-I CHI structures with separation between monocots and dicots. Graphics and alignment were conducted using Jalivew (University of Dundee, United Kingdom).
